# Decision Conflicts in Clinical Care during COVID-19: A Multi-Perspective Inquiry

**DOI:** 10.3390/healthcare10101914

**Published:** 2022-09-29

**Authors:** Joerg Haier, Johannes Beller, Kristina Adorjan, Stefan Bleich, Moritz de Greck, Frank Griesinger, Markus Heppt, René Hurlemann, Soeren Torge Mees, Alexandra Philipsen, Gernot Rohde, Georgia Schilling, Karolin Trautmann, Stephanie E. Combs, Siegfried Geyer, Juergen Schaefers

**Affiliations:** 1 Hannover Medical School, Comprehensive Cancer Center Hannover, 30625 Hannover, Germany; 2 Medical Sociology Unit, Hannover Medical School, 30625 Hannover, Germany; 3 Department of Psychiatry and Psychotherapy, Ludwig-Maximilians-University Hospital, 80539 Munich, Germany; 4 Department of Psychiatry and Psychotherapy, Hannover Medical School, 30625 Hannover, Germany; 5 Department of Psychiatry, Psychosomatic Medicine and Psychotherapy, University Hospital, 60590 Frankfurt am Main, Germany; 6 Department of Hematology and Oncology, Pius-Hospital Oldenburg, Carl von Ossietzky University, 26121 Oldenburg, Germany; 7 Department of Dermatology, Erlangen University Hospital, 91054 Erlangen, Germany; 8 Comprehensive Cancer Center Erlangen-EMN (CCC ER-EMN), 91054 Erlangen, Germany; 9 Department of Psychiatry, Carl von Ossietzky University, 26121 Oldenburg, Germany; 10 Department of General, Visceral and Thoracic Surgery, Friedrichstadt General Hospital, 01067 Dresden, Germany; 11 Department of Psychiatry and Psychotherapy, University Hospital, 53127 Bonn, Germany; 12 Department of Respiratory Medicine and Allergology, University Hospital, Goethe University Frankfurt, 60590 Frankfurt am Main, Germany; 13 Department of Hematology, Oncology, Palliative Care and Rheumatology, Asklepios Tumorzentrum, 22763 Hamburg, Germany; 14 Department of Hematology and Oncology, University Hospital, 01307 Dresden, Germany; 15 Department of Radiation Oncology, Technical University of Munich (TUM), Klinikum rechts der Isar, 81675 Munich, Germany

**Keywords:** decision conflicts, moral distress, uncertainty, oncology, psychiatry, COVID-19

## Abstract

**Background:** The early COVID-19-pandemic was characterized by changes in decision making, decision-relevant value systems and the related perception of decisional uncertainties and conflicts resulting in decisional burden and stress. The vulnerability of clinical care professionals to these decisional dilemmas has not been characterized yet. **Methods:** A cross-sectional questionnaire study (540 patients, 322 physicians and 369 nurses in 11 institutions throughout Germany) was carried out. The inclusion criterion was active involvement in clinical treatment or decision making in oncology or psychiatry during the first year of COVID-19. The questionnaires covered five decision dimensions (conflicts and uncertainty, resources, risk perception, perception of consequences for clinical processes, and the perception of consequences for patients). Data analysis was performed using ANOVA, Pearson rank correlations, and the Chi²-test, and for inferential analysis, nominal logistic regression and tree classification were conducted. **Results:** Professionals reported changes in clinical management (27.5%) and a higher workload (29.2%), resulting in decisional uncertainty (19.2%) and decisional conflicts (22.7%), with significant differences between professional groups (*p* < 0.005), including anxiety, depression, loneliness and stress in professional subgroups (*p* < 0.001). Nominal regression analysis targeting “Decisional Uncertainty” provided a highly significant prediction model (LQ *p* < 0.001) containing eight variables, and the analysis for “Decisional Conflicts” included six items. The classification rates were 64.4% and 92.7%, respectively. Tree analysis confirmed three levels of determinants. **Conclusions:** Decisional uncertainty and conflicts during the COVID-19 pandemic were independent of the actual pandemic load. Vulnerable professional groups for the perception of a high number of decisional dilemmas were characterized by individual perception and the psychological framework. Coping and management strategies should target vulnerability, enable the handling of the individual perception of decisional dilemmas and ensure information availability and specific support for younger professionals.

## 1. Introduction

The COVID-19 pandemic has led to major changes and uncertainties in almost all areas of society. All actors in the healthcare system are still faced with comprehensive challenges, not only due to the global significance of this infectious event but also due to a particularly high level of its complexity. The pandemic is characterized, at least in part, by modulating effects on existing decision-making structures and procedures, as well as decision-relevant value systems. For example, applicable evidence for potential treatment risks within the novel pandemic environment can be largely impaired. In this context, the dynamics have not only revealed a hitherto unimagined need to adapt decision-making algorithms but also shown that numerous changes are not specifically linked to the exponential infection event. The loss of applicable clinical evidence, decisional uncertainty and moral distress have been assumed to interfere with process management in healthcare [1], trust in healthcare structures [2] and patient-related shared decision making (SDM) [3]. Examples include different clinical settings, such as primary care [4], cardiology [5], psychiatry [6] and oncology [1].

Extensive decisional uncertainty during a pandemic can be a result of an inability to handle the extent of variability during decision making due to an increasing lack or impairment of evidence and a rapid increase in additional items that need to be considered. Within this intensively changed environment of clinical care, moral injuries can occur owing to the psychological distress resulting from decisions that cause conflicts with one’s beliefs or values. This dilemma can become critical for patient care during the pandemic [7] and is not limited to emergency care due to infections [8]. Distress in healthcare professionals, as well as patients, due to COVID-19 has also been described in various clinical situations, such as in oncology [9], intensive care [10,11] and geriatrics [12]. In addition, pandemic stigmatization may potentiate impaired healthcare and decision making for structurally vulnerable patient populations, as well as for healthcare professionals [13]. Recently, we reported that specific subgroups of patients can be identified that are characterized by a dedicated vulnerability to this decisional vulnerability and related burden and distress [14]. Similarly, in a regional investigation, we found that about 25% of professionals in cancer care suffer from decisional burden, but the characterization of this group was not included in this smaller trial [1]. However, to our knowledge, comparative and comprehensive investigations of decisional dilemmas in various stakeholder groups have not been published yet.

Multi-stakeholder approaches have been recommended to deal with this dilemma [15]. Intra- and inter-communication between all stakeholders may have a specific role in coping strategies involving all players in the development of adapted processes during the pandemic. However, all of these approaches require an understanding of the underlying stakeholders’ perspectives regarding decisional uncertainties and conflicts. For example, for intensive care, the levels and causes of moral distress have been reported to vary between professional groups and changed during the pandemic [16]. However, a parallel investigation of these decisional perceptions in different stakeholder groups (patients, physicians and nurses) within the same pandemic setting has rarely been conducted. In addition, analytical approaches to identify professional subgroups that show a dedicated vulnerability to this decisional dilemma are currently lacking. Although clinical decisions by various professional groups and patients are made in different backgrounds, the related decisional uncertainty and conflicts deal with the same decisional and clinical processes as part of sufficient SDM. Therefore, it seems to be required to compare the different perspectives of these decisional processes. One major aim of our investigation was to determine whether the perception of decisional dilemmas and pandemic consequences depends on the clinical environment or whether this is an independent phenomenon across all stakeholder groups.

In order to provide guidelines for handling pandemic-related decisional uncertainty, the identification of this specific vulnerability within professional stakeholder groups appears to be required. The characterization of these susceptible (sub)groups can be a basis for the development of coping strategies, supervision programs and educational approaches. The aim of the OnCoVID-1 and -2 studies (“Management in clinical care during the COVID-19 pandemic-ethical, legal, and health economic implications.”) was to identify and characterize professional groups at specific risk for decisional uncertainty and conflicts during the pandemic, as well as factors that modulate this susceptibility. Therefore, we analyzed the individual decisional uncertainties and perceptions of decisional conflicts in clinical processes during the pandemic and potential determinants that can characterize specific pandemic-related decisional vulnerability. Professional groups (nurses and physicians) with immediate involvement in clinical care in oncology and psychiatry were investigated to quantify the extent and areas of conflicts and the determinants and consequences of specific vulnerability and potential protective factors. Furthermore, oncology and psychiatry have a common basis regarding potential decisional dilemmas. In both clinical areas, changes in clinical management may have very severe consequences for patients. Therefore, we compared oncology (potential prognostic consequences) and psychiatry (potentially threatening for coping). Professionals in both clinical areas were chosen as two groups that were assumed to be especially vulnerable to those problems.

## 2. Materials and Methods

### 2.1. Questionnaire Development

As the first step in collecting data, qualitative interviews were performed with physicians, nurses and patients (*n* = 5 in each group) actively involved in cancer or psychiatry care in order to assess their perceptions of decision making during the pandemic and potential consequences for healthcare. The results of the qualitative interviews were used to construct standardized questionnaires. The questionnaires covered 5 decision dimensions (conflicts and uncertainty, resources, risk perception, perception of consequences for clinical processes, and the perception of consequences for patients) that were extracted from the interviews. Every dimension was assessed by 3–5 questions, some of them with questions on detailed aspects of the topics covered. For the different target groups, the questions were slightly adapted according to the respective clinical environment, but an overlap was maintained as much as possible. Since, for this type of questionnaire, standard data for comparison are not applicable, validation is related to the understanding of the intended questions by the participants and the consideration of key aspects of the targeted topic. This was carried out using a Delphi approach (2 rounds of feedback by the target group). Therefore, OnCoVID questionnaires were validated by using 5 representatives of each professional group and patient representatives.

### 2.2. Sample

Cross-sectional data from the OnCoVID study (ethical approval 9199_BO_K_2020) were used. Data were collected through pen and paper surveys between October 2020 and June 2021 from patients, nurses and physicians in 11 participating hospitals (university and non-academic hospitals) throughout Germany. Participating hospitals were selected to cover all major areas of the country. Questionnaire participants were contacted via the cooperating clinics and outpatient centers and invited by mail to participate in the survey. The inclusion criterion was active involvement in the direct treatment of the respective patients and related clinical decision making during the pandemic. Patients had to be receiving active treatment at any stage of their disease.

### 2.3. Variables

Six different versions of the questionnaire specifically adapted for different target groups were used (patients, physicians, nurses in oncology and psychiatry). Overall, 216 different variables were integrated, and 130 (60.2%) of them were applied in multiple target groups (identical questions and answer options), enabling a comparison of the responses (Supplementary Table S1). Decision conflicts were operationalized as previously described [1]. The remaining items were based on similar questions but included group-specific subitems. Briefly, different answer options were “yes” or “no” or on equidistant 5-point scales (from “not at all” to “completely”; “not at all/seldom” to “most of the time”; “much less” to “no changes” to “much more”; “not likely” to “very likely”; and “very negatively” to “no changes” to “very positively”). All of these scales can be considered equidistant. Additional demographic variables were professional experience (measured numerically in years), gender (male or female), specialty (psychiatry or oncology) and stakeholder group (physician, nurse or patient).

### 2.4. Data Analysis

First, the data were analyzed descriptively by designing histograms and boxplots. Items were characterized by mean ± SD and 95% confidence intervals.

For comparisons between the target groups, ANOVA was applied, including Tukey HSD for post hoc tests. Pearson rank order correlations were used to compare groups, and t-tests were performed for continuous variables in the case of two-group comparisons. Chi²-tests with continuity correction were used in the case of categorical variables. Potential determinants of uncertainty and conflicts, as well as potential collinearity, were evaluated by 2-sided Pearson correlations.

For inferential statistical analysis, a two-step approach was applied. First, due to the focus on different stakeholder groups, nominal logistic regression was performed. The identified determinants were used for a subsequent classification tree analysis. As dependent variables, “Decisional Uncertainty” and “Decisional Conflicts” were defined. Non-respondents were excluded pairwise from analyses of the respective items.

For the confirmation of the robustness of the determinants and to enable the differentiated characterization of vulnerability, the obtained regressors were included in a tree classification. As a build-up method, the Chi² automatic interaction detection (CHAID) was used. The number of levels was limited to N = 4, and the minimum size of knots was determined to be N = 50 participants. The significance for splitting was accepted at *p* < 0.005.

All analyses were performed using SPSS26.

## 3. Results

### 3.1. Questionnaire Responses

Overall, N = 1231 (730 females, 473 males, and 28 N/A) questionnaires were returned among the six stakeholder groups (Table 1), representing an adequate response rate of 54.8%. The average age of all participants was 47.4 ± 15.9 years (range 16–67 years). The medical professionals (physicians and nurses) reported 16.4 ± 11.8 years of professional/clinical experience after their professional education (range 0–46 years). A total of 46.6% of the participating physicians were junior residents, 17.4% were consultants, and 27.8% were senior residents/heads of divisions (0.6% N/A). In addition, 23.6% of the nurses were in leading positions. Out of the professional groups, 108 participants (20.0%) had personal experience with the COVID-19 quarantine. Within the patient group, 33.7% received initial treatment, 30.0% continued their previous treatment, 22.2% were treated due to the recurrence of a disease, and 7.8% were in follow-up (6.3% N/A) at the time of the questionnaire.

### 3.2. Decisional Uncertainty and Conflicts

**Figure 1:**
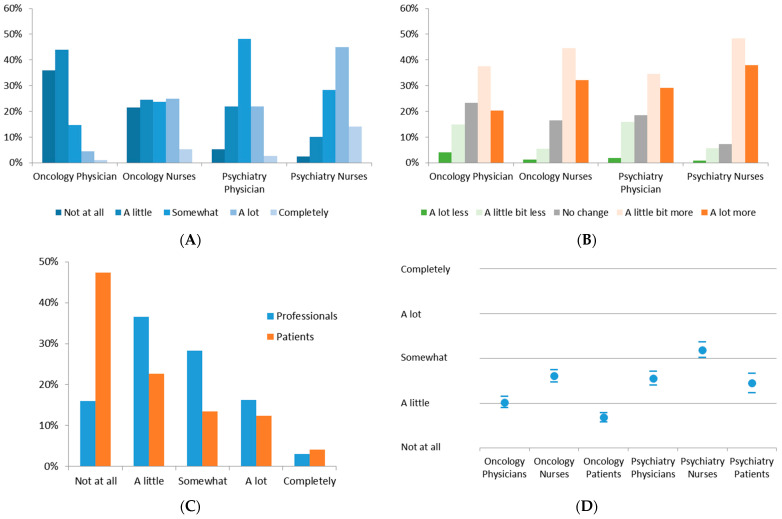
(**A**) Required modifications in clinical management due to pandemic; (**B**) changes in workload due to pandemic situation; (**C**) histogram of uncertainty perception throughout all participating groups; (**D**) decisional uncertainty within the stakeholder groups (mean and 95% CI).

As a result of the pandemic situation, 27.5% of the professionals reported intensive modifications to their clinical decisions (answering with the options “A lot” and “Completely”). The extent of these modifications significantly differed between the various stakeholder groups (*p* < 0.001). The highest values were reported by nurses in psychiatry (3.58 ± 0.94), and the lowest values were reported by physicians in oncology (1.91 ± 0.88) (Figure 1A). This was accompanied by changes in professional workload, which also differed between the professional groups and had higher values for nurses than for physicians (*p* < 0.001) (Figure 1B). Overall, 29.2% of the professionals perceived “A lot more” workload.

Decisional uncertainty was reported in all groups, and all of them showed variability within the spectrum of severity (Figure 1C). Interestingly, 19.2% of the professionals and 16.5% of the patients perceived extensive decisional uncertainty (“A lot” and “Completely”). Surprisingly, patients reflected less intensive decisional uncertainty in both oncology and psychiatry compared to the professional groups. Between the groups, significant differences (*p* < 0.005) were observed, with the lowest perception reported by oncology patients and the highest in the psychiatry nursing group (Figure 1D, Supplementary Table S2). Nurses had the highest perception of uncertainty, and physicians’ as well as patients’ responses were significantly lower (*p* < 0.001). The largest differences were observed between the oncology and psychiatry groups (*p* < 0.001).

Due to the differences between professionals and patients in their perception and the complexity of factors that can potentially influence this reflection, the subsequent analysis was limited to professionals and their decisional uncertainty and conflicts.

**Figure 2:**
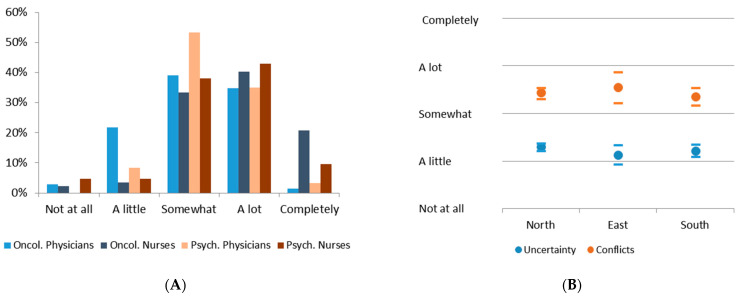
(**A**) Histogram of decisional conflicts within professional groups; (**B**) regional distribution of decisional uncertainty and conflicts in Germany (mean and 95% CI).

Overall, 280 professionals (22.7%) reported their own conflicts in their clinical decisions during the pandemic situation, and for 196 (15.9%), this was directly related to the specific oncological or psychiatric treatment. These participants were questioned as to whether these decisional conflicts resulted in specific burden, and 48.1% of them reported extensive emotional stress (Figure 2). This was similar in all professional groups, and differences were only seen between physicians and oncology nurses (oncology physicians: *p* < 0.001; psychiatry physicians: *p* = 0.027) (Figure 2A).

Initially, we assumed that the extent of the pandemic load, such as incidences and hospitalization rates, determined the intensity of decisional uncertainty and conflicts. Therefore, various regions in Germany (North, East and South) that had suffered from the pandemic to very different extents at the time of the questionnaire were compared. Surprisingly, such differences were not observed in either category (Figure 2B). It is remarkable that the perceived decisional conflicts were, on average, more than one scaling category higher compared to decisional uncertainty, although this difference was not significant due to the high distribution of the reported intensity. Both individual reflections of the decisional dilemma correlated significantly with each other (*p* < 0.001; Pearson R = 0.324).

### 3.3. Consequences of Decisional Uncertainty and Conflicts

**Figure 3:**
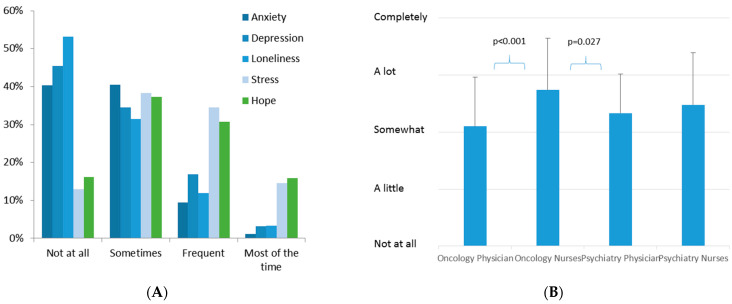
(**A**) Histograms of personal conditions for professionals. (**B**) Burden due to decisional conflicts in different professional groups.

Anxiety, depression, loneliness and stress were reported by professionals, and large subgroups answered “Frequent” or “Most of the time” (Figure 3A). However, almost half of both stakeholder groups also reported hope to the same extent. The occurrence of decisional uncertainty and resulting distress were highly correlated with this perception by professionals (Supplementary Table S3).

When the “Burden due to Decisional Conflicts” question was analyzed, significantly higher values were seen in the nursing group compared to physicians (*p* < 0.001), but there were no significant differences between oncology and psychiatry professionals (*p* = 0.649). In a multivariate analysis of this item, oncology nurses suffered from a significantly higher burden than oncology (*p* < 0.001) and psychiatry physicians (*p* = 0.027) (Figure 3B).

### 3.4. Reasons and Vulnerability

**Figure 4:**
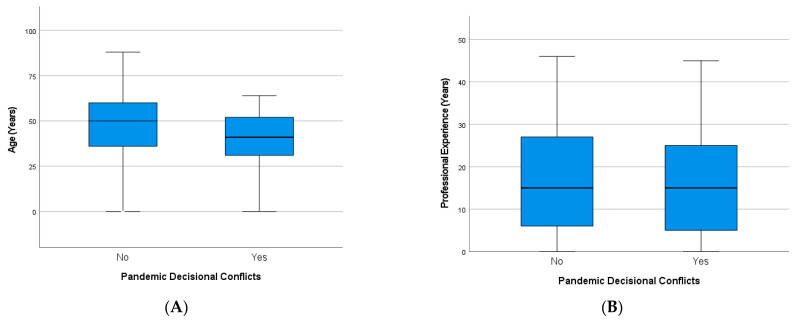
Factors of vulnerability to decisional conflicts of healthcare professionals during the pandemic: (**A**) age (*p* < 0.001) and (**B**) professional experience (n.s.).

Professionals who reported decisional conflicts were significantly younger than those without conflicts (*p* < 0.001), but their professional experience was not significantly different (Figure 4). This was similar in both professional groups (not shown).

Nominal regression analysis targeting “Decisional Uncertainty” provided a highly significant prediction model (Likelihood–Quotient Test *p* < 0.001). Pseudo-R² (Nagelkerke = 0.657) also confirmed high model quality. The resulting regression coefficients were significant or nearly significant for eight parameters (Table 2A). The variable “Gender” was not significant and was excluded from further modeling. The resulting classification showed 61.3% correct predictions on the 5-point scale. If the prediction of neighboring categories was also considered acceptable, 92.5% of the participants were sufficiently classified using these parameters (Table 2B).

In a similar approach, “Decisional Conflicts” were evaluated. The Likelihood–Quotient Test *p* < 0.001) and Pseudo-R² (Nagelkerke = 0.620) supported high model quality. However, the prediction was achieved with only six parameters that were equally found in the parameter list for “Decisional Uncertainty”. The items “Gender” and “Burden due to Distress” were not significant predictors for this variable (Table 3A). The classification rates were 64.4% and 92.7%, respectively (Table 3B).

**Figure 5:**
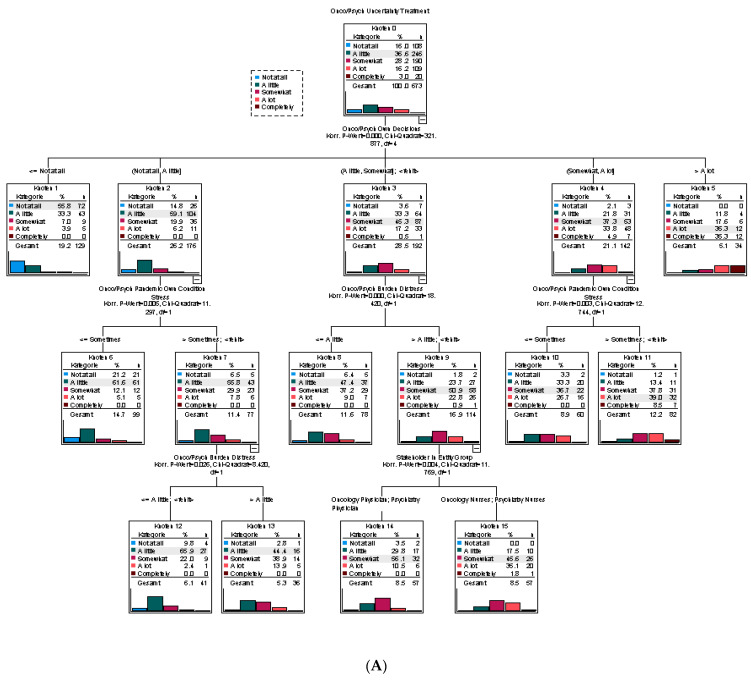

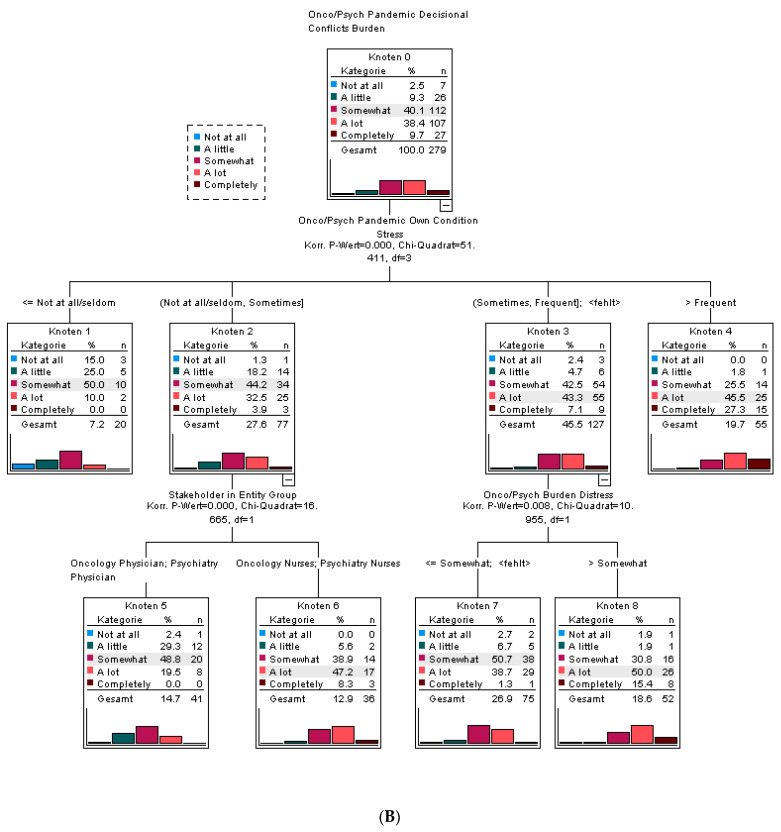
Tree classification for parameters influencing (**A**) “Decisional Uncertainty” and (**B**) “Decisional Conflicts”.

Finally, we performed tree analyses to further characterize the uncertainty and conflicts during the pandemic. Parameters that were predictors in the regression analyses were used to model the tree classification. The obtained tree for “Decisional Uncertainty” showed that the most important predictor was the requirement to modify one’s own clinical decisions. If these modifications did not occur in the individual’s own clinical environment, uncertainty was mainly absent. In contrast, once modifications were required “A lot” or “Completely”, very high uncertainty was predicted. In the middle range of uncertainty, the presence of the individual’s stress and their own burden due to pandemic-related distress were related to more intense uncertainty. An influence of the professional group (physicians versus nurses) was only seen with a minor impact, whereas gender, age, the perception of one’s own risk and information deficit were not included (Figure 5A).

A tree analysis for “Decisional Conflicts” was performed on the 279 professionals with a complete dataset who reported decisional conflicts. The minimum group size was reduced to 30 per knot. This resulted in only three levels but with different involvement of the regressors compared to “Decisional Uncertainty”. The most important factor was the existence of “Stress due to the pandemic” for the professionals. The absence of stress resulted in the lowest levels of conflicts, and very high stress levels (>“Frequent”) were solely predictive of decisional conflicts. Within stress group 2 (“Sometimes”), physicians had less conflict compared to nurses. Stress group 3 (“Frequent”) was differentiated by the participants’ own burden due to distress (Figure 5B). If a larger minimum group size per knot was chosen (N = 50), this “Burden due to distress” gained even more importance as a predictor (data not shown). Surprisingly, the requirement to “Modify own clinical decisions”, the “Pandemic workload”, the “Burden due to Own Risk” and the age of the participants did not contribute as determinants of classification levels.

## 4. Discussion

Decisional uncertainty and decisional conflicts largely affect clinical care. However, various stakeholders and different specialties have been assumed to be involved to various extents. Based on previous investigations, it was expected that the pandemic increased decisional issues to a large extent and caused moral distress for all stakeholder groups. The questionnaires in our investigation were designed to capture the individual perspectives and perceptions of the participants’ special situation during the early phase of the pandemic and not objective data, such as caseload, infection rates and guideline adherence, among others.

Our data demonstrate that this decisional dilemma was of high importance in all investigated groups, and up to one-third of the healthcare professionals suffered from intensive uncertainty and conflicts due to the COVID-19 situation. Although we did not have comparable data before and after the onset of the pandemic, the design of the questions enabled us to conclude that both uncertainty and conflicts increased to a large extent because of COVID-19. We designed the questions in a manner that specifically addressed the perception (not objective process data) of pandemic consequences and decisional dilemmas due to COVID-19. This needs to be differentiated from the objective impacts of the pandemic on clinical processes. Our approach seems to be especially feasible for the analysis of individual perceptions of pandemic consequences. If questions are designed in such a way, they allow the analysis of individual consequences. In this regard, our results underline the importance of differentiating between objective impacts on clinical processes (meso- or macro-level) and individual perceptions (micro-level) [17].

Similar to our quantitative data, Austin et al. [18], in a small qualitative study, reported the conflicting feelings of female healthcare professionals while providing care, managing information and decisions, and balancing roles, coping and well-being. Also comparable to our investigation, high stress levels in 32% of general practitioners were reported by Dutour et al. [19].

It was very surprising that we could not find differences between various regions in Germany, although they had very large differences in their pandemic involvement during the time of questionnaire administration. There appears to be a dissociation between objective changes in treatment organization due to pandemic conditions and individual perceptions of their consequences for healthcare. This was furthermore independent of the age and gender of the professionals. Since we included two very different specialties, it seems to be reasonable to consider this a general pandemic-related effect. This is supported by our finding that the perceived changes in professional workload due to the pandemic situation were a predictor of both aspects, but its impact was lower compared to other identified determinants of this vulnerability. In a small study involving healthcare workers in breast cancer care, Vanni et al. [20] also found that pandemic-induced stress was not related to age, gender, workload or the level of pandemic involvement. In contrast, gender differences were observed for healthcare leadership by Luoto and Varella [21] and Bacigalupe et al. [22], but this was not in a clinical context and not supported by empirical data.

Another, at least in part, unexpected observation was the fact that healthcare professionals reported, on average, higher values for uncertainty and conflicts than patients who were treated by these professionals. Related differences between nurses and physicians were indeed expected and might be caused by a different understanding of clinical aspects or closer contact with patients, among others. Similar to our previous result that psycho-oncology was the most problematic challenge in oncology during the first period of the pandemic [1], psychiatry, with its strong dependence on direct patient communication, was more involved in perceived decisional dilemmas than professionals working in oncology.

Overall, decisional conflicts were reported, on average, at higher levels compared to decisional uncertainty. The high variability and extent of the decisional dilemma in larger professional groups led to the question of whether this vulnerability can be characterized. The similarity throughout Germany and the dissociation between objective alterations in the professional environment under pandemic conditions led to the assumption that individual coping strategies, antagonizing factors and the psychological environments of professionals may act as determinants of the vulnerability regarding this dilemma. Using interferential statistics, gender and the duration of professional experience were excluded as relevant determinants for decisional problems. Individual risk perception plays a role in decision making to handle the pandemic crisis, but similar to our results, only moderate impacts have been published for other countries [23]. The most important factors in our study were the individual’s own involvement in treatment modifications due to the pandemic, professionals’ own stress levels and the individual burden caused by the pandemic situation. Although significant in the regression analysis by age, the availability of information and the professional group had only minor impacts on the classification of vulnerability. The insufficient availability of information as a key determinant was confirmed in another investigation [19]. In contrast to our findings, the importance of perceived physicians’ expertise was described by Martínez-Sanz et al. [24] to attenuate their tolerance for uncertainty.

Overall, our results suggest that vulnerability to decisional dilemmas is mainly determined by the direct involvement of the deficiency of evidence of impaired opportunities to perform regular treatment due to the pandemic, with a resulting necessity for modifications in healthcare processes. It seems to be potentiated by the resulting stress and the individual perception of burden. Additional workload and the objective impacts of the pandemic are negligible for this vulnerability, and we found strong evidence for the dissociation between the perception and objective indicators of the pandemic. Protective factors were identified as older age and the availability of information, both underlining the importance of the individual decisional framework. Increased intrinsic motivation to deal with this healthcare crisis [25] or sociocultural aspects [26] may also influence the extent of the decisional dilemma during the pandemic, but this was not operationalized in our investigation.

The relationship between the perception of uncertainty and decisional conflicts may be influenced by additional factors related to healthcare professionals and/or their environments that were not included in this investigation. For example, the specificity of the social roles of each stakeholder group, the availability of individual coping strategies, self-efficacy, the level of mutual trust and the individual social status are additional potential determinants of this perception. In the case of nurses, this may be attributed to their decreased freedom of choice compared to physicians, increased reliance on doctors’ decisions and their overall lower status, which contribute to a high stress load.

The inclusion of a high number of centers throughout Germany representing very different involvement in the pandemic at the time of data collection is a strength of this investigation: in addition, the wide coverage of potential determinants of decisional burden is unique to our investigation. Therefore, this study was able to perform a multivariate approach for the identification of their specific roles. In our investigation, we did not use standard tools to capture stress levels and decisional uncertainty. In our opinion, those questionnaires were developed for an environment that is only partially comparable to a pandemic crisis. Furthermore, none of these instruments could include pandemic-specific topics. Therefore, we implemented and validated a unique questionnaire for all relevant stakeholder groups for clinical care that was validated during the implementation of the study. Due to the mail-based acquisition of participants, we cannot completely rule out a selection bias within the respondents, but sufficient response rates likely reduced this problem.

Further analysis is required to evaluate the vulnerability of patients regarding the decisional dilemma and to compare the different stakeholders’ perspectives on the perception of pandemic-induced modifications in healthcare.

## 5. Conclusions

Decisional uncertainty and decisional conflicts due to the very specific situation of a pandemic, together with the resulting moral distress for healthcare professionals, likely had a high impact on the quality of patient care and safety. Since a multi-strategy approach needs to be considered for various stakeholders to enable sustainable working conditions during and beyond this pandemic [27], uncertainty and decisional conflicts constitute a very serious risk factor for occupational burnout. Therefore, the proposed actions should also include extensive burnout prevention among vulnerable groups. Coping and management strategies should account for the vulnerability to these decisional issues and need to consider strengthening individual armamentaria to deal with the situation, ensuring the best availability of information and specific support for younger healthcare professionals.

## Figures and Tables

**Table 1:** Numbers of respondents within the stakeholder groups.

	# Respondents	Female/Male ^a^	Age ^b^	Experience ^c^	% Respond
Oncology Physicians	207	97/109	43.3 ± 10.3	15.2 ± 10.5	16.8
Oncology Nurses	241	202/35	42.3 ± 12.3	19.1 ± 12.3	19.6
Oncology Patients	386	197/183	59.7 ± 15.6		31.4
Psychiatry Physicians	115	63/51	38.5 ± 10.5	9.9 ± 9.2	9.3
Psychiatry Nurses	128	85/33	43.4 ± 19.3	19.3 ± 12.6	10.4
Psychiatry Patients	154	86/62	39.3 ± 15.6		12.5
Total	1231	730/473	47.4 ± 15.9	16.4 ± 11.8	100.0

^a^ Twenty-eight participants were excluded due to lack of information; ^b^ years (mean ± SD); ^c^ years of professional/clinical experience after professional education (mean ± SD); **#** Number Respondents.

**Table 2:** Prediction by nominal regression analysis for “Decisional Uncertainty”: (A) Likelihood–Quotient Test for regression parameters and (B) resulting classification.

**(A)**							
**Effect**				**−2 Log-Likelihood for Reduced Model**	**Likelihood-Quotient Tests**		
					**Chi-Square**	**dF**	**Significance**
Constant term				1086.079	0.000	0	0.000
Stakeholder in entity group				1111.436	25.357	12	0.013
Age				1094.640	8.562	4	0.073
Gender				1101.177	15.098	12	0.236
Availability of professional information				1120.034	33.955	16	0.006
Modification of own decisions				1262.868	176.789	16	0.000
Burden due to distress				1598.556	512.477	16	0.000
Burden due to own risk				1110.867	24.788	16	0.074
Own condition stress				1120.687	34.608	12	0.001
Pandemic workload				1112.688	26.609	16	0.046
**(B)**							
**Observed**	**Predicted**						
	**Not at All**	**A Little**	**Somewhat**	**A Lot**	**Completely**	**% Correct**	**% Correct with Neighbor**
Not at all	55	35	5	0	0	57.9%	94.7%
A little	24	137	41	11	3	63.4%	93.5%
Somewhat	4	37	113	12	1	67.7%	97.0%
A lot	2	16	29	40	2	44.9%	79.8%
Completely	0	0	2	2	13	76.5%	88.2%
% Total	14.6%	38.5%	32.5%	11.1%	3.3%	61.3%	92.5%

**Table 3:** Prediction by nominal regression analysis for “Decisional Conflicts”: (A) Likelihood–Quotient Test for regression parameters and (B) resulting classification.

**(A)**							
**Effect**				**−2 Log-Likelihood for Reduced Model**	**Likelihood-Quotient Tests**		
					**Chi-Square**	**dF**	**Significance**
Constant term				428.226	0.000	0	0.000
Age				1101.176	672.950	4	0.000
Gender				434.342	6.117	8	0.634
Stakeholder in entity group				515.481	87.256	12	0.000
Modification of own decisions				500.931	72.705	16	0.000
Burden due to own risk				526.510	98.284	16	0.000
Burden due to distress				442.304	14.078	16	0.593
Own condition stress				502.116	73.891	12	0.000
Pandemic workload				511.219	82.993	16	0.000
**(B)**							
**Observed**	**Predicted**						
	**Not at All**	**A Little**	**Somewhat**	**A Lot**	**Completely**	**% Correct**	**% Correct with Neighbor**
Not at all	4	0	1	1	0	66.7%	66.7%
A little	0	11	6	8	0	44.0%	68.0%
Somewhat	0	3	72	24	2	71.3%	98.0%
A lot	0	2	28	55	4	61.8%	97.8%
Completely	0	0	4	5	17	65.4%	84.6%
% Total	1.6%	6.5%	44.9%	37.7%	9.3%	64.4%	92.7%

## Data Availability

The data that support the findings of this study are available on request from the corresponding author. The data are not publicly available due to privacy or ethical restrictions.

## Supplementary Materials

The following supporting information can be downloaded at: https://www.mdpi.com/article/10.3390/healthcare10101914/s1, Suppl. Table S1: List of items captured in the OnCoVID questionnaires. Questions were used for either oncology, psychiatry or both specialties (onco/psych). Questions for professionals and patients with the same content were merged for evaluation, if applicable. Suppl. Table S2: Significant differences in the 5-point scale of decisional uncertainty between stakeholder groups. Multivariate comparison was performed using Tukey HSD test. * Significant differences *p* < 0.05. Suppl. Table S3: Correlations of items reflecting the psychological environment of healthcare professions. Pearson correlation, 2-sided significance and numbers of included respondents. ** Significant correlations *p* < 0.01.
