# Supplementary material for: Decision Conflicts in Clinical Care during COVID-19: A Multi-Perspective Inquiry
Source: Healthcare (Basel). 2022 Sep 29;10(10):1914. doi: 10.3390/healthcare10101914 (PMC9602416; doi:10.3390/healthcare10101914)
Supplement: Supplementary file 1 [file healthcare-10-01914-s001.zip › Suppl. Table S1.pdf]

Decision Conflicts in Oncological Care During COVID-19: A Multi-Perspective Inquiry

Suppl. Table S1: List of items captured in the OnCoVID-questionnaires. Questions were either used for oncology, psychiatry or both specialties (onco/psych). Questions for professionals and patients with the same content were merged for evaluation, if applicable.

ID Questionnaire

Stakeholder Group

Speciality Group

Stakeholder in Entity Group

Oncology Treatment Modification Screening

Oncology Treatment Modification Curative Drug

Oncology Treatment Modification Curative Surgery

Oncology Treatment Modification Advanced Drug

Oncology Treatment Modification Advanced Surgery

Oncology Treatment Modification Radiooncology

Oncology Treatment Modification Palliative

Oncology Nursing Modification Requirement

Oncology Nursing Modification Advice

Oncology Nursing Modification Relationship

Oncology Nursing Modification Psychosocial

Oncology Nursing Modification Relatives

Oncology Nursing Modification Treatment Assistance

Oncology Nursing Modification Terminal Care

Oncology Nursing Consequences Requirement

Oncology Nursing Consequences Advice

Oncology Nursing Consequences Relationship

Oncology Nursing Consequences Psychosocial

Oncology Nursing Consequences Relatives

Oncology Nursing Consequences Treatment Assistance

Oncology Nursing Consequences Terminal Care

Oncology Treatment Consequences Screening

Oncology Treatment Consequences Curative Drug

Oncology Treatment Consequences Curative Surgery

Oncology Treatment Consequences Advanced Drug

Oncology Treatment Consequences Advanced Surgery

Oncology Treatment Consequences Radiooncology

Oncology Treatment Consequences Palliative

Oncology General Consequences Prevention

Oncology General Consequences Curative

Oncology General Consequences Advanced

Oncology General Consequences Palliative

Oncology General Consequences Psychosocial

Psychiatry Treatment Modification Psychiatry

Psychiatry Treatment Modification Psychotherapy

Psychiatry Treatment Modification Ergotherapy

Psychiatry Treatment Modification Physiotherapy

Psychiatry Treatment Modification Sociotherapy

Psychiatry Treatment Modification Accomodation

Psychiatry Treatment Consequences Psychiatry

Psychiatry Treatment Consequences Psychotherapy

Psychiatry Treatment Consequences Ergotherapy

Psychiatry Treatment Consequences Physiotherapy

Psychiatry Treatment Consequences Sociotherapy

Psychiatry Treatment Consequences Accomodation

Psychiatry General Consequences Inpatient

Psychiatry General Consequences Daycare

Psychiatry General Consequences Outpatient

Psychiatry General Consequences Homecare

Psychiatry General Consequences Accomodation

Onco/Psych Own Decisions

Oncology Decision Criteria Overall Survival

Oncology Decision Criteria Eventfree Survival

Psychiatry Decision Criteria Suicidality

Psychiatry Decision Criteria Reccurence

Onco/Psych Decision Criteria Symptoms

Onco/Psych Decision Criteria Side Effects/Complications

Onco/Psych Decision Criteria SARS Additional Risk

Onco/Psych Uncertainty Treatment

Onco/Psych Legal Obligation

Onco/Psych Management General

Onco/Psych Management Quality

Onco/Psych Management Hygiene

Onco/Psych Management Data Protection

Onco/Psych Management Trials

Onco/Psych Management Informed Consent

Onco Nursing Management Multiprofessional Exchange

Onco/Psych Distance Regulations

Onco/Psych Ressources Staff

Oncology Ressources Surgery

Oncology Ressources Radiooncology

Onco/Psych Ressources Drug Treatment

Onco/Psych Ressources Drug Availability

Onco/Psych Ressources Diagnostics

Oncology Ressources Palliative

Onco/Psych Ressources Protective

Psychiatry Ressources Beds

Psychiatry Ressources Psychotherapy

Onco/Psych Processes Referral To Inpatient

Onco/Psych Processes Referral To Outpatient

Onco/Psych Processes Homecare

Onco/Psych Processes Social Support

Onco/Psych Processes Psycho(onco)logical Support

Oncology Processes Second Opinion

Onco/Psych Processes Follow Up

Oncology Processes Terminal Care

Psychiatry Processes Socialpsychiatry

Onco/Psych Nursing Processes Observation

Onco/Psych Nursing Processes Care

Onco/Psych Nursing Referral

Psychiatry Masks Impairment Treatment

Psychiatry Masks Impairment Diagnostics

Onco/Psych Burden Professional Groups

Onco/Psych Burden Patients

Onco/Psych Burden Communication

Onco/Psych Burden Uncertainty

Onco/Psych Burden Distress

Onco/Psych Burden Testing

Onco/Psych Burden Own Risk

Onco/Psych Information Own Entity

Onco/Psych Information External Professional

Onco/Psych Information Professional Education

Onco/Psych Pandemic Workload

Onco/Psych Pandemic Decisional Conflicts

Onco/Psych Pandemic Decisional Conflicts Context

Onco/Psych Pandemic Decisional Conflicts Burden

Onco/Psych Patient Treatment Uncertainty

Onco/Psych Patient Uncertainty Distress

Onco/Psych Pandemic Own Risk

Onco/Psych Patient Pandemic Own Risk

Onco/Psych Pandemic Own Condition Anxiety

Onco/Psych Pandemic Own Condition Depression

Onco/Psych Pandemic Own Condition Loneliness

Onco/Psych Pandemic Own Condition Hope

Onco/Psych Pandemic Own Condition Stress

Sociodemographic Postal Code

Sociodemographic Age

Sociodemographic Gender

Sociodemographic Function

Sociodemographic Experience

Sociodemographic Professional Field

Sociodemographic Quarantine

Oncology Patient Treatment Modification Screening

Oncology Patient Treatment Modification Drug

Oncology Patient Treatment Modification Surgery

Oncology Patient Treatment Modification Radiooncology

Oncology Patient Treatment Modification Other Treatment

Oncology Patient Treatment Modification Follow up

Oncology Patient Treatment Modification Psychosocial

Oncology Patient General Consequences Screening

Oncology Patient General Consequences Tumorspecific

Oncology Patient General Consequences Follow up

Oncology Patient General Consequences Psychosocial

Oncology Patient General Consequences Rehabilitation

Oncology Patient Treatment Consequences Screening

Oncology Patient Treatment Consequences Drug

Oncology Patient Treatment Consequences Radiooncology

Oncology Patient Treatment Consequences Follow up

Oncology Patient Treatment Consequences Psychosocial

Oncology Patient Treatment Consequences Rehabilitation

Oncology Patient Treatment Consequences Other Treatment

Onco/Psych Patient Own Decisions

Onco/Psych Patient Decision Support Physician

Onco/Psych Patient Decision Support Social Environment

Onco/Psych Patient Decision Support Own Evaluation

Onco/Psych Patient Decision Criteria Treatment Response

Onco/Psych Patient Decision Criteria Symptoms

Onco/Psych Patient Decision Criteria Side Effects/Complications

Onco/Psych Patient Decision Criteria SARS Additional Risk

Onco/Psych Patient Professional Attention Physicians

Onco/Psych Patient Professional Attention Nurses

Oncology Patient Professional Attention Other Professions

Onco/Psych Patient Professional Attention Government

Psychiatry Patient Professional Attention Psychologists

Psychiatry Patient Professional Attention Ergotherapists

Psychiatry Patient Professional Attention Physiotherapists

Psychiatry Patient Professional Attention Sociotherapists

Onco/Psych Patient Distance Regulations

Onco/Psych Patient Burden Professional Groups Physicians

Psychiatry Patient Burden Professional Groups Psychologists

Onco/Psych Patient Burden Professional Groups Nurses

Onco/Psych Patient Burden Professional Groups Others

Onco/Psych Patient Relationship Professional Groups Physicians

Psychiatry Patient Relationship Professional Groups Psychologists

Onco/Psych Patient Relationship Professional Groups Nurses

Onco/Psych Patient Relationship Professional Groups Others

Onco/Psych Patient Communication Professional Groups Physicians

Psychiatry Patient Communication Professional Groups Psychologists

Onco/Psych Patient Communication Professional Groups Nurses

Onco/Psych Patient Communication Professional Groups Others

Onco/Psych Patient Uncertainty Physicians

Psychiatry Patient Uncertainty Psychologists

Onco/Psych Patient Uncertainty Nurses

Onco/Psych Patient Uncertainty Others

Onco/Psych Patient Distress Physicians

Psychiatry Patient Distress Psychologists

Onco/Psych Patient Distress Nurses

Onco/Psych Patient Distress Others

Onco/Psych Patient Treatment Location Change

Onco/Psych Patient Own SARS Test Performance

Onco/Psych Patient Own SARS Test Expectation

Onco/Psych Patient Burden Infection Risk

Onco/Psych Patient Information Availability Own Provider

Onco/Psych Patient Information Availability External

Onco/Psych Patient Contact SHG

Onco/Psych Patient Contact SHG Change

Onco/Psych Patient Education

Onco/Psych Patient Disease Stage

Psychiatry Patient Treatment Modification Conversational Therapy

Psychiatry Patient Treatment Modification Drug

Psychiatry Patient Treatment Modification Ergotherapy

Psychiatry Patient Treatment Modification Physiotherapy

Psychiatry Patient Treatment Modification Sociotherapy

Psychiatry Patient Treatment Modification Follow up

Psychiatry Patient Treatment Modification Rehabilitation

Psychiatry Patient Treatment Modification SHG

Psychiatry Patient General Consequences Conversational Therapy

Psychiatry Patient General Consequences Drug

Psychiatry Patient General Consequences Ergotherapy

Psychiatry Patient General Consequences Physiotherapy

Psychiatry Patient General Consequences Sociotherapy

Psychiatry Patient General Consequences Follow up

Psychiatry Patient General Consequences Rehabilitation

Psychiatry Patient General Consequences SHG

Psychiatry Patient Treatment Consequences Conversational Therapy

Psychiatry Patient Treatment Consequences Drug

Psychiatry Patient Treatment Consequences Ergotherapy

Psychiatry Patient Treatment Consequences Physiotherapy

Psychiatry Patient Treatment Consequences Sociotherapy

Psychiatry Patient Treatment Consequences Follow up

Psychiatry Patient Treatment Consequences Rehabilitation

Psychiatry Patient Treatment Consequences SHG
