# Supplementary material for: Decision Conflicts in Clinical Care during COVID-19: A Multi-Perspective Inquiry
Source: Healthcare (Basel). 2022 Sep 29;10(10):1914. doi: 10.3390/healthcare10101914 (PMC9602416; doi:10.3390/healthcare10101914)
Supplement: Supplementary file 1 [file healthcare-10-01914-s001.zip › Suppl. Tables S2 and S3.pdf]

Decision Conflicts in Oncological Care During COVID-19: A Multi-Perspective Inquiry

Suppl. Table S2: Significant differences at 5-point scale of decisional uncertainty between stakeholder groups. Multivariate comparison was done using Tukey-HSD test. * Significant differences $p < 0.05$.

					95% Confidence interval	
	Stakeholder in Entity Group	Mean differences	Std.-Div.	Sig.	Lower level	Upper level
Oncology Physician	Oncology Nurses	−0.589*	0.097	0.000	−0.87	−0.31
	Oncology Patient	0.349*	0.088	0.001	0.10	0.60
	Psychiatry Physician	−0.538*	0.119	0.000	−0.88	−0.20
	Psychiatry Nurses	−1.167*	0.115	0.000	−1.49	−0.84
	Psychiatry Patient	−0.417*	0.110	0.002	−0.73	−0.10
Oncology Nurses	Oncology Physician	0.589*	0.097	0.000	0.31	0.87
	Oncology Patient	0.938*	0.085	0.000	0.70	1.18
	Psychiatry Physician	0.051	0.116	0.998	−0.28	0.38
	Psychiatry Nurses	−0.578*	0.112	0.000	−0.90	−0.26
	Psychiatry Patient	0.172	0.107	0.588	−0.13	0.48
Oncology Patient	Oncology Physician	−0.349*	0.088	0.001	−0.60	−0.10
	Oncology Nurses	−0.938*	0.085	0.000	−1.18	−0.70
	Psychiatry Physician	−0.887*	0.109	0.000	−1.20	−0.58
	Psychiatry Nurses	−1.516*	0.105	0.000	−1.81	−1.22
	Psychiatry Patient	−0.765*	0.099	0.000	−1.05	−0.48
Psychiatry Physician	Oncology Physician	0.538*	0.119	0.000	0.20	0.88
	Oncology Nurses	−0.051	0.116	0.998	−0.38	0.28
	Oncology Patient	0.887*	0.109	0.000	0.58	1.20
	Psychiatry Nurses	−0.630*	0.131	0.000	−1.00	−0.26
	Psychiatry Patient	0.121	0.127	0.932	−0.24	0.48
Psychiatry Nurses	Oncology Physician	1.167*	0.115	0.000	0.84	1.49
	Oncology Nurses	0.578*	0.112	0.000	0.26	0.90
	Oncology Patient	1.516*	0.105	0.000	1.22	1.81
	Psychiatry Physician	0.630*	0.131	0.000	0.26	1.00
	Psychiatry Patient	0.751*	0.123	0.000	0.40	1.10
Psychiatry Patient	Oncology Physician	0.417*	0.110	0.002	0.10	0.73
	Oncology Nurses	−0.172	0.107	0.588	−0.48	0.13
	Oncology Patient	0.765*	0.099	0.000	0.48	1.05
	Psychiatry Physician	−0.121	0.127	0.932	−0.48	0.24
	Psychiatry Nurses	−0.751*	0.123	0.000	−1.10	−0.40

Suppl. Table S3: Correlations of items reflecting the psychological environment of the healthcare professions. Pearson correlation. 2-sided significance and numbers of included respondents. ** Significant correlations p<0.01

	Anxiety	Depression	Loneliness	Hope	Stress	Treatment Uncertainty
Depression	0.433**		0.534**	−0.179**	0.487**	0.293**
	0.000		0.000	0.000	0.000	0.000
	677		673	652	679	662
Loneliness	0.294**	0.534**		−0.105**	0.291**	0.240**
	0.000	0.000		0.007	0.000	0.000
	671	673		647	673	656
Hope	−0.041	−0.179**	−0.105**		−0.194**	−0.057
	0.293	0.000	0.007		0.000	0.154
	650	652	647		652	636
Stress	0.323**	0.487**	0.291**	−0.194**		0.344**
	0.000	0.000	0.000	0.000		0.000
	677	679	673	652		662
Treatment Uncertainty	0.275**	0.293**	0.240**	−0.057	0.344**	
	0.000	0.000	0.000	0.154	0.000	
	660	662	656	636	662	
Burden by Decisional Conflicts	0.299**	0.393**	0.233**	−0.020	0.414**	0.324**
	0.000	0.000	0.000	0.748	0.000	0.000
	266	267	264	259	267	270
